# Supplementary material for: Comparative Effectiveness of Western and Eastern Manual Therapies for Chronic Obstructive Pulmonary Disease: A Systematic Review and Network Meta-Analysis
Source: Healthcare (Basel). 2021 Aug 30;9(9):1127. doi: 10.3390/healthcare9091127 (PMC8467771; doi:10.3390/healthcare9091127)
Supplement: Supplementary file 1 [file healthcare-09-01127-s001.zip › Supplement S1.pdf]

Supplement S1. Search terms used in each

database Medline via PubMed

	Searches	Results
#1	″Lung Diseases, Obstructive″[MH] OR (obstruct*[TIAB] AND (pulmonary[TIAB] OR airway*[TIAB] OR lung*[TIAB] OR airflow*[TIAB] OR bronch*[TIAB] OR respirat*[TIAB])) OR emphysema[TIAB] OR COPD[TIAB] OR COAD[TIAB] OR COBD[TIAB] OR AECB[TIAB]	301,719
#2	″Musculoskeletal Manipulations″[MH] OR Chiropractic[MH] OR ″manual therapy″[TIAB] OR manipulate*[TIAB] OR ″joint mobilization″[TIAB] OR ″joint mobilisation″[TIAB] OR chiropract*[TIAB] OR osteopath*[TIAB] OR massage[TIAB] OR reflexology[TIAB] OR ″soft tissue therapy″[TIAB] OR ″Muscle Stretching Exercises″[MH] OR ″muscle stretching″[TIAB] OR tuina[TIAB] OR chuna[TIAB] OR ″tui na″[TIAB] OR acupressure[TIAB]	96,927
#3	“Randomized Controlled Trial”[PT] OR “Controlled Clinical Trial”[PT] OR randomized[TIAB] OR placebo[TIAB] OR “Clinical Trials as Topic”[Mesh: noexp] OR randomly[TIAB] OR trial[TI]	1,433,018
#4	animals[MH] NOT humans[MH]	4,788,192
#5	#1 AND #2 AND #3 NOT #4	**92**

EMBASE via Elsevier

	Searches	Results
#1	′chronic obstructive lung disease′/exp OR ′chronic obstructive lung disease′:ab,ti OR (obstruct*:ab,ti AND (pulmonary:ab,ti OR airway*:ab,ti OR lung*:ab,ti OR airflow*:ab,ti OR bronch*:ab,ti OR respirat*:ab,ti)) OR emphysema/exp OR emphysema:ab,ti OR COPD:ab,ti OR COAD:ab,ti OR COBD:ab,ti OR AECB:ab,ti	302,585
#2	‘musculoskeletal manipulation’/exp OR ‘musculoskeletal manipulation’:ab,ti OR chiropractic/exp OR chiropractic:ab,ti OR ‘manipulative medicine’/exp OR ‘manipulative medicine’:ab,ti OR ‘manual therapy’:ab,ti OR ‘joint mobilization’/exp OR ‘joint mobili*’:ab,ti OR ‘osteopathic medicine’/exp OR osteopath*:ab,ti OR massage:ab,ti OR reflexology/exp OR reflexology:ab,ti OR ‘soft tissue therapy’:ab,ti OR ‘stretching exercise’/exp OR ‘muscle stretching’/exp OR ‘muscle stretching′:ab,ti OR tuina:ab,ti OR chuna:ab,ti OR ′tui na′:ab,ti OR acupressure/exp OR acupressure:ab,ti	62,057
#3	'crossover procedure':de OR 'double-blind procedure':de OR 'randomized controlled trial':de OR 'single-blind procedure':de OR (random* OR factorial* OR crossover* OR cross NEXT/1 over* OR placebo* OR doubl* NEAR/1 blind* OR singl* NEAR/1 blind* OR assign* OR allocat* OR volunteer*):de,ab,ti	2,698,374
#4	#1 AND #2 AND #3	**122**

CENTRAL

	Searches	Results
#1	MeSH descriptor: [Lung Diseases, Obstructive] explode all trees	19,769
#2	((obstruct* AND (pulmonary OR airway* OR lung* OR airflow* OR bronch* OR respirat*)) OR emphysema OR COPD OR COAD OR COBD OR AECB):ti,ab,kw	30,528
#3	MeSH descriptor: [Musculoskeletal Manipulations] explode all trees	2,933
#4	MeSH descriptor: [Chiropractic] explode all trees	130
#5	MeSH descriptor: [Muscle Stretching Exercises] explode all trees	609
#6	("manual therapy" OR manipulate* OR "joint mobilization" OR "joint mobilisation" OR chiropract* OR osteopath* OR massage OR reflexology OR "soft tissue therapy" OR "muscle stretching" OR tuina OR chuna OR "tui na" OR acupressure):ti,ab,kw	13,728
#7	((#1 OR #2) AND (#3 OR #4 OR #5 OR #6)) in Trials	**133**

AMED via EBSCO

	Searches	Results
#1	″Lung Diseases, Obstructive″[SU] OR (obstruct*[TX] AND (pulmonary[TX] OR airway*[TX] OR lung*[TX] OR airflow*[TX] OR bronch*[TX] OR respirat*[TX])) OR emphysema[TX] OR COPD[TX] OR COAD[TX] OR COBD[TX] OR AECB[TX]	2,536
#2	″Musculoskeletal Manipulations″[SU] OR Chiropractic[SU] OR ″manual therapy″[TX] OR manipulate*[TX] OR ″joint mobilization″[TX] OR ″joint mobilisation″[TX] OR chiropract*[TX] OR osteopath*[TX] OR massage[TX] OR reflexology[TX] OR ″soft tissue therapy″[TX] OR ″Muscle Stretching Exercises″[SU] OR ″muscle stretching″[TX] OR tuina[TX] OR chuna[TX] OR ″tui na″[TX] OR acupressure[TX]	19,626
#3	#1 AND #2	**60**

KISS

	Searches	Results
#1	(COPD OR 만성 폐쇄성 폐질환) AND (수기요법 OR 관절가동술 OR chiropractic OR 마사지 OR 반사요법)	0
#2	(COPD OR 만성 폐쇄성 폐질환) AND (근육스트레칭 OR 추나 OR 지압)	0
#3	#1 OR #2	**0**

KCI

	Searches	Results
#1	(COPD OR 만성 폐쇄성 폐질환) AND (수기요법 OR 관절가동술 OR chiropractic)	0
#2	(COPD OR 만성 폐쇄성 폐질환) AND (마사지 OR 반사요법 OR 근육스트레칭)	0
#3	(COPD OR 만성 폐쇄성 폐질환) AND (추나 OR 지압)	0
#4	#1 OR #2 OR #3	**0**

CNKI

	Searches	Results
#1	(SU='COPD'+'慢性阻塞性肺疾病'+'慢性阻塞性肺病'+'慢性阻塞性肺'+'肺气肿'+'慢性支气管炎'+'慢性气管炎'+'慢阻肺疾病'+'慢阻肺病'+'慢阻肺') AND (SU=′手法治疗′+′推拿′+′理疗′+′按摩′+′物理疗法′+′捏脊′+′正骨′+′整骨′+′整脊′+′理筋′+′反射疗法′+′肌肉能量技术′+′等距′+′关节松动′+′筋膜松动′+′贴压′+′敷贴′)	**385**

Wanfang data

	Searches	Results
#1	(主题:COPD or 主题:慢性阻塞性肺疾病 or 主题:慢性阻塞性肺病 or 主题:慢性阻塞性肺 or 主题:肺气肿 or 主题:慢性支气管炎 or 主题:慢性气管炎 or 主题:慢阻肺疾病 or 主题:慢阻肺病 or 主题:慢阻肺) AND (主题:手法治疗 or 主题:推拿 or 主题:理疗 or 主题:按摩 or 主题:物理疗法 or 主题:捏脊 or 主题:正骨 or 主题:整骨 or 主题:整脊 or 主题:理筋 or 主题:反射疗法 or 主题:肌肉能量技术 or 主题:等距 or 主题:关节松动 or 主题:筋膜松动 or 主题:贴压 or 主题:敷贴)	**1382**

VIP.

	Searches	Results
#1	(M=(COPD OR 慢性阻塞性肺疾病 OR 慢性阻塞性肺病 OR 慢性阻塞性肺 OR 肺气肿 OR 慢性支气管炎 OR 慢性气管炎 OR 慢阻肺疾病 OR 慢阻肺病 OR 慢阻肺) AND M=(手法治疗 OR 推拿 OR 理疗 OR 按摩 OR 物理疗法 OR 捏脊	**449**

	OR 正骨 OR 整骨 OR 整脊 OR 理筋 OR 反射疗法 OR 肌肉能量技术 OR 等距 OR 关节松动 OR 筋膜松动 OR 贴压 OR 敷贴))	
