# Supplementary material for: Comparative Effectiveness of Western and Eastern Manual Therapies for Chronic Obstructive Pulmonary Disease: A Systematic Review and Network Meta-Analysis
Source: Healthcare (Basel). 2021 Aug 30;9(9):1127. doi: 10.3390/healthcare9091127 (PMC8467771; doi:10.3390/healthcare9091127)
Supplement: Supplementary file 1 [file healthcare-09-01127-s001.zip › Supplement S2.pdf]

Supplement S2. List of excluded studies after full-text review

1) only abstract available with no details: 8

1. 孙武权. 内功推拿治疗慢阻肺研究. 中国民族医药学会第二届全国推拿学术交流会论文集; 郑州2016. p. 19-29.
2. 唐秀光, 李小媛, 陈林. 综合康复改善慢性阻塞性肺病患者生活质量与再发间隔期的临床研究. 第八次全国老年医学学术会议论文集; 厦门2007. p. 99-101.
3. 龚海蓉, 陈锦秀. 足反射区疗法对稳定期COPD患者生活质量和肺功能的影响. 全国中医、中西医结合护理学术交流会议论文汇编; 中国新疆乌鲁木齐2011.
4. Zanotti E, Bizzarri C, Manstretta A, Fracchia C. Osteopathic manipulative treatment effectiveness in chronic obstructive pulmonary disease (COPD). European respiratory society annual congress, barcelona, spain, september 18-22. 2010:[P609].
5. Valderramas S, Sossai L, Marcovic M. Respiratory muscle stretching in patients with chronic obstructive pulmonary disease submitted to a pulmonary rehabilitation program. European respiratory society annual congress, vienna, austria, september 12-16. 2009:[E4304].
6. Gorska K, Maskey-Warzechowska M, Mierzejewski M, Krenke R. Immediate effect of osteopathic manual therapy on hyperinflation in patients with severe chronic obstructive pulmonary disease. European Respiratory Journal. 2017;50.
7. Engel RM, Gonski P, Beath K, Vemulpad S. The inclusion of manual therapy in a pulmonary rehabilitation program for chronic obstructive pulmonary disease: A randomized controlled pilot trial. Respirology. 2013;18:124.
8. Çirak Y, Yelvar GDY, Demir YP, Dalkilin¸ M, Kizilirmak D, Bozkurt B. Effects of additional manual therapy over inspiratory muscle training in patients with chronic obstructive pulmonary disease: A randomized controlled trial. European Respiratory Journal. 2015;46.

2) case series: 1

1. 高弘, 孙晓莉. 物理疗法、腹式呼吸训练治疗慢性阻塞性肺病. 现代康复. 2000(05):776.

3) quasi-RCTs: 4

1. 盛海燕, 郭秀君. 震动排痰仪联合穴位按摩在COPD痰浊阻肺症患者中的应用. 齐鲁护理杂志. 2017;23(19):105-6.
2. 盛海燕, 郭秀君. 震动排痰仪联合穴位按摩治疗慢性阻塞性肺病患者排痰疗效比较. 大家

健康（中旬版）. 2016;10(9):118-.
3. 盛海燕. 震动排痰仪联合穴位按摩对慢性阻塞性肺病患者排痰效果的临床研究 [硕士]: 南京中医药大学; 2017.
4. 李双. 中医康复及护理对COPD患者肺功能与焦虑抑郁心理情绪的影响分析. 临床医药文献电子杂志. 2019;6(41):99.

4) review articles: 2

1. 丁建新. 慢性阻塞性肺疾病患者的康复治疗. 新医学. 1999;30(8).
2. Galletti J, McHeileh G, Hahne A, Lee AL. The clinical effects of manipulative therapy in people with chronic obstructive pulmonary disease. Journal of Alternative and Complementary Medicine. 2018;24(7):677-83.

5) not meeting intervention criteria: 10

1. 裴燕, 王晔琳, 瞿真. 穴位按摩配合氧驱动雾化吸入治疗AECOPD的临床疗效观察. 临床合理用药杂志. 2016;9(04):106-7.
2. Quanqing M. The study of long term curative effect of chronic obstructive pulmonary disease in remission stage treated with TCM. Pakistan journal of pharmaceutical sciences. 2017;30(3):1121-4.
3. Mehl-Madrona L, Kligler B, Silverman S, Lynton H, Merrell W. The impact of acupuncture and craniosacral therapy interventions on clinical outcomes in adults with asthma. Explore (New York, NY). 2007;3(1):28-36.
4. 李冬庆. 治疗慢性阻塞性肺疾病64例临床疗效观察. 健康必读. 2018(36):256.
5. 陈静, 李淑杏, 陈长香, 王烁烁. 综合穴位按摩对改善COPD病人认知功能和生活质量的效果研究. 护理研究. 2019;33(04):660-2.
6. 郭娟, 陀健林, 农美蓉, 卢茵. 穴位按摩在慢性阻塞性肺疾病患者家庭护理中的作用. 中西医结合护理(中英文). 2017;3(09):93-5.
7. Maa SH, Sun MF, Hsu KH, Hung TJ, Chen HC, Yu CT, et al. Effect of Acupuncture or Acupressure on Quality of Life of Patients with Chronic Obstructive Asthma: A Pilot Study. Journal of Alternative and Complementary Medicine. 2003;9(5):659-70.
8. Maa SH, Gauthier D, Turner M. Acupressure as an adjunct to a pulmonary rehabilitation program. Journal of Cardiopulmonary Rehabilitation. 1997;17(4):268-76.
9. Maa S. Acupressure for COPD patients. European respiratory journal - supplement. 1998;12(Suppl 28):25S.

10. Noll DR, Johnson JC, Baer RW, Snider EJ. The immediate effect of individual manipulation techniques on pulmonary function measures in persons with chronic obstructive pulmonary disease. Osteopathic medicine and primary care. 2009;3:9.

6) without outcomes of interest: 5

1. 周旭玲. 物理疗法在COPD护理中的应用. 护士进修杂志. 2005(08):729-30.
2. 张晨旭, 陈卓, 罗熙, 吕蕾, 贺资淋. 体位引流联合穴位按摩治疗慢性阻塞性肺病患者排痰疗效比较. 东方药膳. 2020(11):128.
3. 潘分乔, 李振联, 侯小藏. 穴位按摩治疗慢性支气管炎迁延期58例疗效观察. 河北中医. 2010;32(05):725-6.
4. Rocha HM, Muniz de Souza HC, Viana R, Neves VR, Dornelas de Andrade A. Immediate Effects of Rib Mobilization and Diaphragm Release Techniques on Cardiac Autonomic Control in Patients With Chronic Obstructive Pulmonary Disease: A Pilot Study. Journal of chiropractic medicine. 2020;19(3):167-74.
5. Ayrapetova NS, Eremushkin MA, Antonovich IV, Kuznetsov OF, Samorukov AE, Budylin SP, et al. The rehabilitative treatment of the patients presenting with chronic obstructive pulmonary disease including the application of the manual handling methods. Voprosy kurortologii, fizioterapii, i lechebnoi fizicheskoi kultury. 2017;94(3):11-6.

7) using duplicated data: 4

1. 高丽云, 盛海燕, 朱永磊. 机械辅助排痰联合穴位按摩在痰浊壅肺型慢性阻塞性肺疾病病人护理中的应用. 护理研究. 2017;31(02):182-5.
2. Gao L, Sheng H, Zhu Y. Application of mechanical-assisted expectoration combined with acupoint massage in nursing care of phlegm obstructing lung type of chronic obstructive pulmonary disease patients. Chinese nursing research. 2017;31(2):182-5.
3. Cross J, Elender F, Barton G, Clark A, Shepstone L, Blyth A, et al. A randomised controlled equivalence trial to determine the effectiveness and cost-utility of manual chest physiotherapy techniques in the management of exacerbations of chronic obstructive pulmonary disease (MATREX). Health technology assessment (Winchester, England). 2010;14(23):1-147, iii-iv.
4. Chen Q, Zhong LW, Liu HB, Zhang JF, Xie GG, Jin XQ, et al. Massage therapy for chronic obstructive pulmonary disease. Chinese Journal of Clinical Rehabilitation. 2006;10(7):10-2.

8) unavailable full-text: 2

1. 朱莲莲. 穴位按摩对慢性阻塞性肺病的观察效果. 养生保健指南：医药研究. 2015;0(12).

2. Masarsky CS, Weber M. Chiropractic management of chronic obstructive pulmonary disease. Journal of Manipulative and Physiological Therapeutics. 1988;11(6):505-10.
