# Supplementary material for: Comparative Effectiveness of Western and Eastern Manual Therapies for Chronic Obstructive Pulmonary Disease: A Systematic Review and Network Meta-Analysis
Source: Healthcare (Basel). 2021 Aug 30;9(9):1127. doi: 10.3390/healthcare9091127 (PMC8467771; doi:10.3390/healthcare9091127)
Supplement: Supplementary file 1 [file healthcare-09-01127-s001.zip › Supplement S3_revised.pdf]

Supplement S3. Results of pairwise meta-analysis

1. FEV1 (L)

	Experimental			Control				Mean Difference	Risk of Bias						
Study or Subgroup	Mean	SD	Total	Mean	SD	Total	Weight	IV, Random, 95% CI	A	B	C	D	E	F	G
**1.1.1 MAN+ROC vs. Sham+ROC**															
Noll 2008	1.18	0.62	18	1.28	63	17	0.0%	-0.10 [-30.05, 29.85]	?	?	−	+	+	+	+
Zanotti 2012	1.13	0.4	10	0.9	0.4	10	100.0%	0.23 [-0.12, 0.58]	+	?	+	+	+	+	+
**Subtotal (95% CI)**			**28**			**27**	**100.0%**	**0.23 [-0.12, 0.58]**							
Heterogeneity: Tau² = 0.00; Chi² = 0.00, df = 1 (P = 0.98); I² = 0%															
Test for overall effect: Z = 1.29 (P = 0.20)															
**1.1.2 MAS+ROC vs. ROC**															
Kurzaj 2013	1.4	0.26	20	1.4	0.7	10	48.6%	0.00 [-0.45, 0.45]	?	?	?	?	+	+	+
Zhuang 2017	2.901	0.09	35	1.569	0.256	35	51.4%	1.33 [1.24, 1.42]	?	?	?	?	+	+	+
**Subtotal (95% CI)**			**55**			**45**	**100.0%**	**0.68 [-0.62, 1.99]**							
Heterogeneity: Tau² = 0.86; Chi² = 32.56, df = 1 (P < 0.00001); I² = 97%															
Test for overall effect: Z = 1.03 (P = 0.30)															
**1.1.3 ACU+ROC vs. ROC**															
Guo 2017	1.2	0.57	100	1.06	0.53	100	34.5%	0.14 [-0.01, 0.29]	+	?	?	?	+	+	−
Huang 2018	1.885	0.531	34	2.216	0.538	34	29.5%	-0.33 [-0.59, -0.08]	?	?	?	?	+	+	+
Zhang 2020	1.85	0.29	45	1.57	0.26	45	36.0%	0.28 [0.17, 0.39]	?	?	?	?	+	+	+
**Subtotal (95% CI)**			**179**			**179**	**100.0%**	**0.05 [-0.24, 0.34]**							
Heterogeneity: Tau² = 0.06; Chi² = 18.66, df = 2 (P < 0.0001); I² = 89%															
Test for overall effect: Z = 0.35 (P = 0.73)															
**1.1.4 TUI+ROC vs. ROC**															
Chen 2006	1.419	0.953	15	1.333	0.798	15	5.7%	0.09 [-0.54, 0.72]	?	?	?	?	+	+	+
Zhang 2005	1.6	0.29	31	1.5	0.39	32	78.8%	0.10 [-0.07, 0.27]	+	?	?	?	−	−	+
Zhang 2015	1.417	0.951	40	1.331	0.788	40	15.4%	0.09 [-0.30, 0.47]	?	?	?	?	+	+	+
**Subtotal (95% CI)**			**86**			**87**	**100.0%**	**0.10 [-0.05, 0.25]**							
Heterogeneity: Tau² = 0.00; Chi² = 0.01, df = 2 (P = 1.00); I² = 0%															
Test for overall effect: Z = 1.26 (P = 0.21)															
**1.1.5 MANP+ROC vs. MECP+ROC**															
Wang 2009	2	0.74	60	2.48	0.74	60	100.0%	-0.48 [-0.74, -0.22]	?	?	?	?	+	+	+
**Subtotal (95% CI)**			**60**			**60**	**100.0%**	**-0.48 [-0.74, -0.22]**							
Heterogeneity: Not applicable															
Test for overall effect: Z = 3.55 (P = 0.0004)															

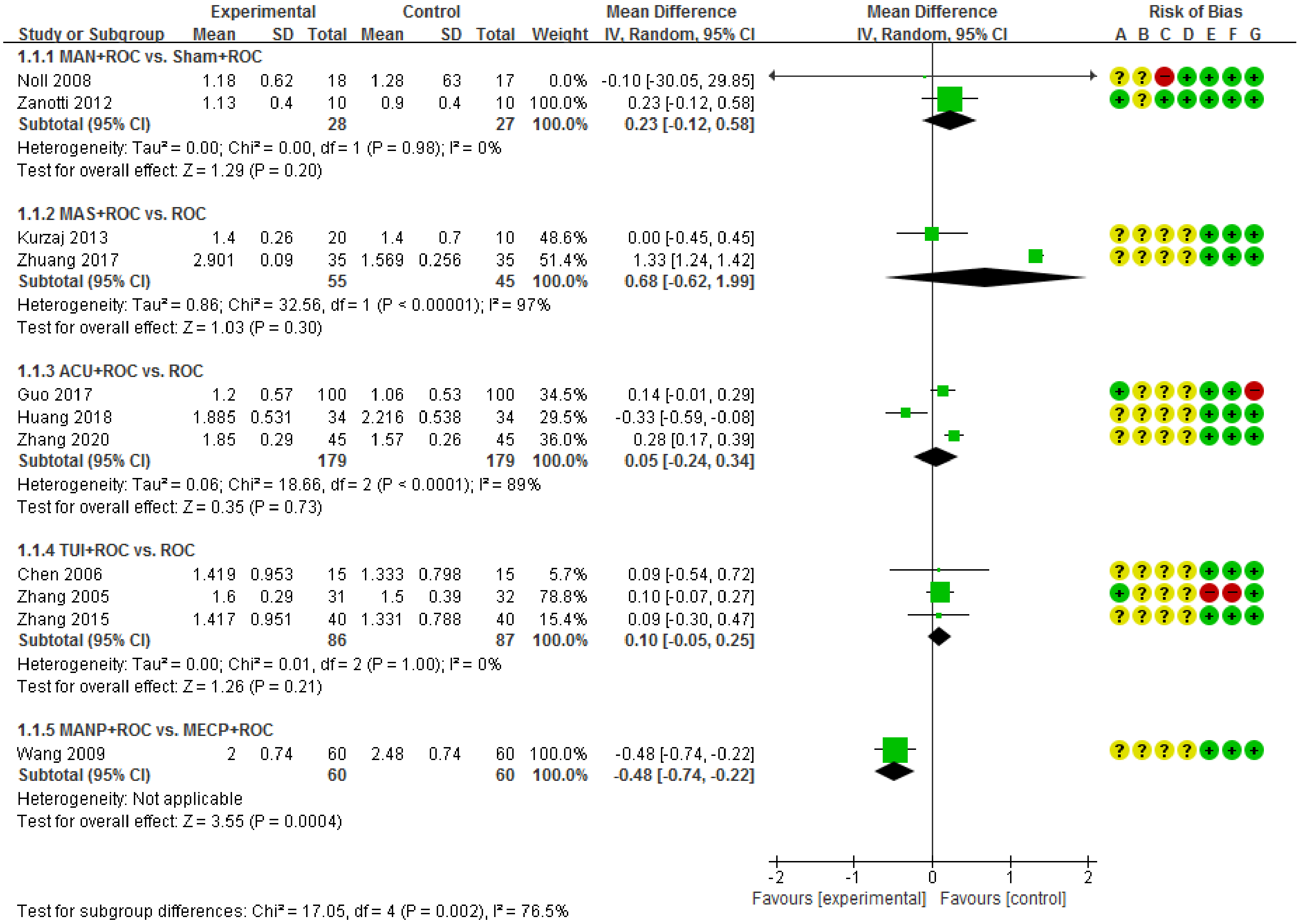

Test for subgroup differences: Chi² = 17.05, df = 4 (P = 0.002), I² = 76.5%

Risk of bias legend
(A) Random sequence generation (selection bias)
(B) Allocation concealment (selection bias)
(C) Blinding of participants and personnel (performance bias)
(D) Blinding of outcome assessment (detection bias)
(E) Incomplete outcome data (attrition bias)
(F) Selective reporting (reporting bias)
(G) Other bias

2. FVC (L)

Study or Subgroup	Experimental Mean	Experimental SD	Experimental Total	Control Mean	Control SD	Control Total	Weight	Mean Difference IV, Random, 95% CI	A	B	C	D	E	F	G
**1.3.1 MAN+ROC vs. Sham+ROC**															
Noll 2008	2.36	0.93	18	2.66	0.92	17	50.3%	-0.30 [-0.91, 0.31]	?	?	−	+	+	+	+
Zanotti 2012	2.05	0.6	10	1.79	0.8	10	49.7%	0.26 [-0.36, 0.88]	+	?	+	+	+	+	+
**Subtotal (95% CI)**			**28**			**27**	**100.0%**	**-0.02 [-0.57, 0.53]**							
Heterogeneity: Tau² = 0.06; Chi² = 1.59, df = 1 (P = 0.21); I² = 37%															
Test for overall effect: Z = 0.08 (P = 0.94)															
**1.3.2 ACU+ROC vs. ROC**															
Zhang 2020	2.58	0.41	45	2.25	0.34	45	100.0%	0.33 [0.17, 0.49]	?	?	?	?	+	+	+
**Subtotal (95% CI)**			**45**			**45**	**100.0%**	**0.33 [0.17, 0.49]**							
Heterogeneity: Not applicable															
Test for overall effect: Z = 4.16 (P < 0.0001)															
**1.3.3 TUI+ROC vs. ROC**															
Chen 2006	2.628	0.921	15	2.362	0.759	15	26.9%	0.27 [-0.34, 0.87]	?	?	?	?	+	+	+
Zhang 2015	2.625	0.931	40	2.361	0.729	40	73.1%	0.26 [-0.10, 0.63]	?	?	?	?	+	+	+
**Subtotal (95% CI)**			**55**			**55**	**100.0%**	**0.26 [-0.05, 0.58]**							
Heterogeneity: Tau² = 0.00; Chi² = 0.00, df = 1 (P = 1.00); I² = 0%															
Test for overall effect: Z = 1.65 (P = 0.10)															
**1.3.4 MANP+ROC vs. MECP+ROC**															
Wang 2009	3.06	0.78	60	3.45	0.75	60	100.0%	-0.39 [-0.66, -0.12]	?	?	?	?	+	+	+
**Subtotal (95% CI)**			**60**			**60**	**100.0%**	**-0.39 [-0.66, -0.12]**							
Heterogeneity: Not applicable															
Test for overall effect: Z = 2.79 (P = 0.005)															

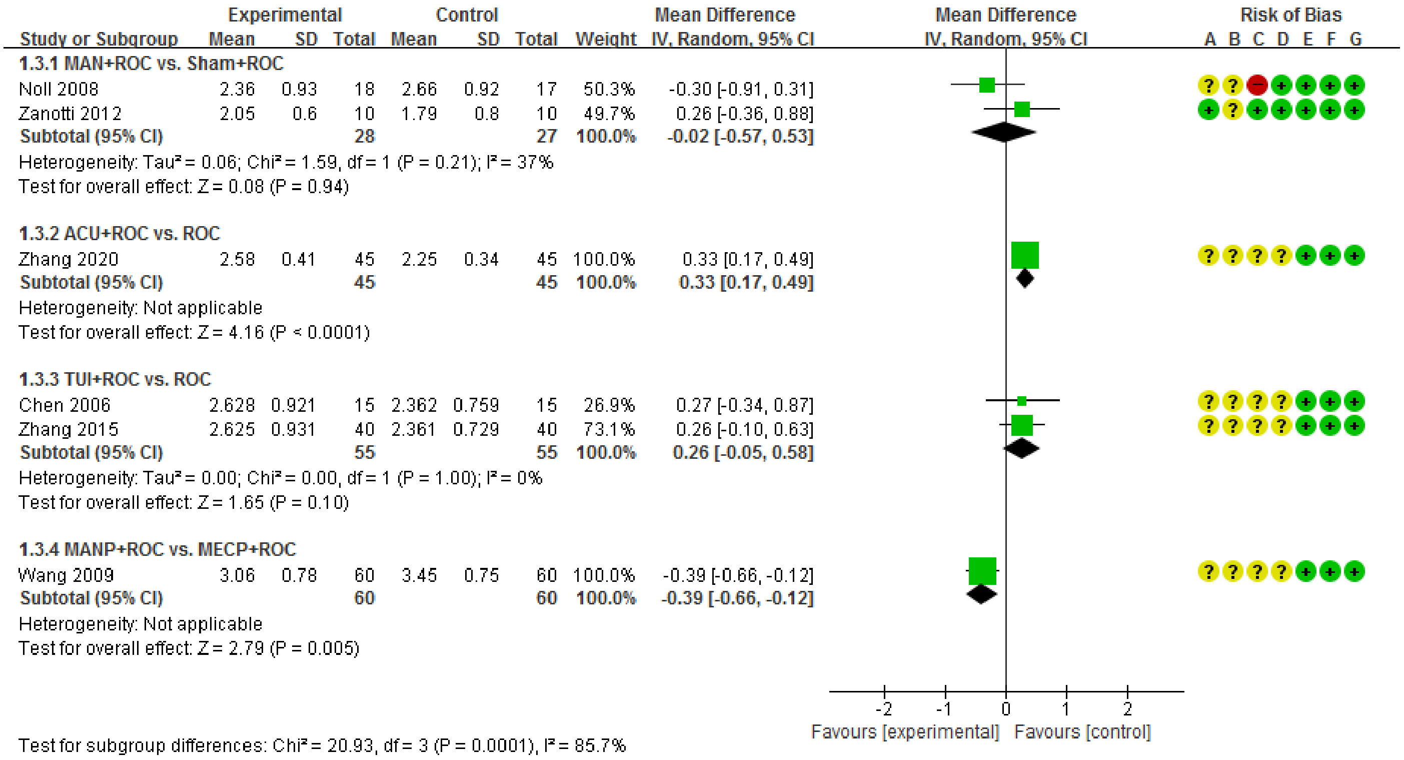

Test for subgroup differences: Chi² = 20.93, df = 3 (P = 0.0001), I² = 85.7%

Risk of bias legend
(A) Random sequence generation (selection bias)
(B) Allocation concealment (selection bias)
(C) Blinding of participants and personnel (performance bias)
(D) Blinding of outcome assessment (detection bias)
(E) Incomplete outcome data (attrition bias)
(F) Selective reporting (reporting bias)
(G) Other bias

3. FEV1/FVC (%)

Study or Subgroup	Experimental Mean	Experimental SD	Experimental Total	Control Mean	Control SD	Control Total	Weight	Mean Difference IV, Random, 95% CI	A	B	C	D	E	F	G
**1.5.1 MAN+ROC vs. Sham+ROC**															
Noll 2008	48.89	12.88	18	45.88	16.66	17	100.0%	3.01 [-6.90, 12.92]	?	?	-	+	+	+	+
**Subtotal (95% CI)**			**18**			**17**	**100.0%**	**3.01 [-6.90, 12.92]**							
Heterogeneity: Not applicable															
Test for overall effect: Z = 0.60 (P = 0.55)															
**1.5.2 MAS+ROC vs. ROC**															
Zhuang 2017	80.54	9.69	35	60.54	9.69	35	100.0%	20.00 [15.46, 24.54]	?	?	?	?	+	+	+
**Subtotal (95% CI)**			**35**			**35**	**100.0%**	**20.00 [15.46, 24.54]**							
Heterogeneity: Not applicable															
Test for overall effect: Z = 8.63 (P < 0.00001)															
**1.5.3 ACU+ROC vs. ROC**															
Guo 2017	46.9	13.61	100	51.71	15.4	100	32.9%	-4.81 [-8.84, -0.78]	+	?	?	?	+	+	-
Huang 2018	52.109	6.711	34	50.091	6.085	34	35.7%	2.02 [-1.03, 5.06]	?	?	?	?	+	+	+
Zhang 2020	60.96	11.48	45	55.54	10.54	45	31.4%	5.42 [0.87, 9.97]	?	?	?	?	+	+	+
**Subtotal (95% CI)**			**179**			**179**	**100.0%**	**0.84 [-4.60, 6.27]**							
Heterogeneity: $Tau^2 = 19.16$; $Chi^2 = 12.02$, df = 2 (P = 0.002); $I^2 = 83\%$															
Test for overall effect: Z = 0.30 (P = 0.76)															
**1.5.4 TUI+ROC vs. ROC**															
Chen 2006	54.57	11.25	15	50.6	9.62	15	11.6%	3.97 [-3.52, 11.46]	?	?	?	?	+	+	+
Zhang 2005	61.4	6.65	31	59.8	7.08	32	56.8%	1.60 [-1.79, 4.99]	+	?	?	?	-	-	+
Zhang 2015	54.55	11.15	40	50.5	9.52	40	31.6%	4.05 [-0.49, 8.59]	?	?	?	?	+	+	+
**Subtotal (95% CI)**			**86**			**87**	**100.0%**	**2.65 [0.10, 5.20]**							
Heterogeneity: $Tau^2 = 0.00$; $Chi^2 = 0.85$, df = 2 (P = 0.65); $I^2 = 0\%$															
Test for overall effect: Z = 2.03 (P = 0.04)															

Mean Difference
IV, Random, 95% CI

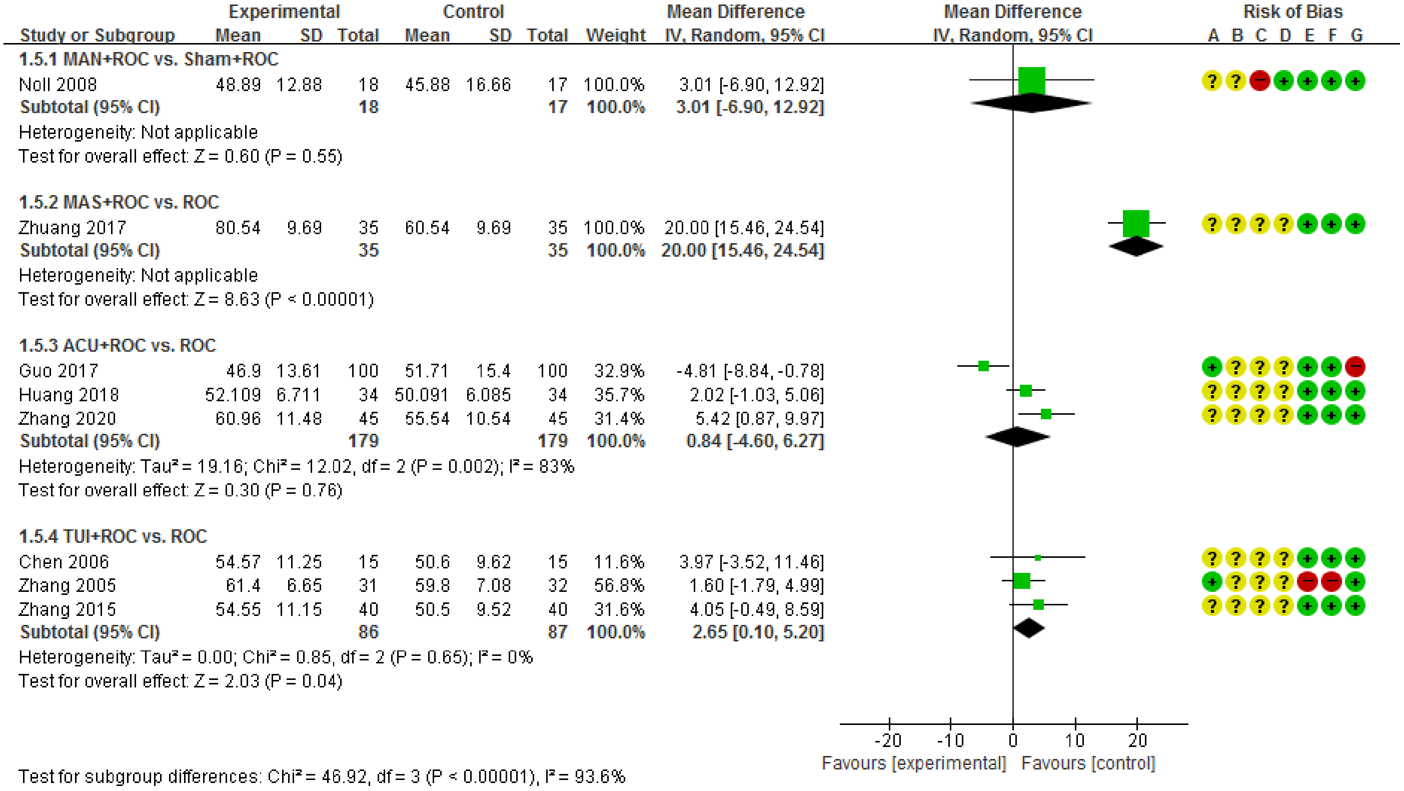

Test for subgroup differences: $Chi^2 = 46.92$, df = 3 (P < 0.00001), $I^2 = 93.6\%$

Risk of bias legend

(A) Random sequence generation (selection bias)
(B) Allocation concealment (selection bias)
(C) Blinding of participants and personnel (performance bias)
(D) Blinding of outcome assessment (detection bias)
(E) Incomplete outcome data (attrition bias)
(F) Selective reporting (reporting bias)
(G) Other bias

4. 6MWD (m)

Study or Subgroup	Experimental Mean	Experimental SD	Experimental Total	Control Mean	Control SD	Control Total	Weight	Mean Difference IV, Random, 95% CI	Risk of Bias A	B	C	D	E	F	G
**1.6.1 MAN+ROC vs. Sham+ROC**															
Zanotti 2012	369.5	80	10	304.7	96.6	10	100.0%	64.80 [-12.94, 142.54]	+	?	+	+	+	+	+
**Subtotal (95% CI)**			**10**			**10**	**100.0%**	**64.80 [-12.94, 142.54]**							
Heterogeneity: Not applicable															
Test for overall effect: Z = 1.63 (P = 0.10)															
**1.6.2 MAS+ROC vs. ROC**															
Kurzaj 2013	318.7	73.6	20	262.5	89.9	10	100.0%	56.20 [-8.18, 120.58]	?	?	?	?	+	+	+
**Subtotal (95% CI)**			**20**			**10**	**100.0%**	**56.20 [-8.18, 120.58]**							
Heterogeneity: Not applicable															
Test for overall effect: Z = 1.71 (P = 0.09)															
**1.6.3 ACU+ROC vs. ROC**															
Huang 2018	384.38	21.08	34	370	23.74	34	100.0%	14.38 [3.71, 25.05]	?	?	?	?	+	+	+
**Subtotal (95% CI)**			**34**			**34**	**100.0%**	**14.38 [3.71, 25.05]**							
Heterogeneity: Not applicable															
Test for overall effect: Z = 2.64 (P = 0.008)															
**1.6.4 TUI+ROC vs. ROC**															
Chen 2006	389.73	72.15	15	346.65	69.23	15	16.8%	43.08 [-7.52, 93.68]	?	?	?	?	+	+	+
Mo 2016	448.4	62.8	29	380.5	45.6	28	40.3%	67.90 [39.48, 96.32]	?	?	?	?	+	+	+
Zhang 2005	398	58.33	31	363.2	50.77	32	42.9%	34.80 [7.76, 61.84]	+	?	?	?	−	−	+
**Subtotal (95% CI)**			**75**			**75**	**100.0%**	**49.53 [27.05, 72.00]**							
Heterogeneity: Tau² = 116.16; Chi² = 2.81, df = 2 (P = 0.25); I² = 29%															
Test for overall effect: Z = 4.32 (P < 0.0001)															
**1.6.5 FRF+ROC vs. ROC**															
Huang 2017	302.31	59.05	29	266.23	48.52	30	100.0%	36.08 [8.45, 63.71]	+	?	?	?	+	+	+
**Subtotal (95% CI)**			**29**			**30**	**100.0%**	**36.08 [8.45, 63.71]**							
Heterogeneity: Not applicable															
Test for overall effect: Z = 2.56 (P = 0.01)															

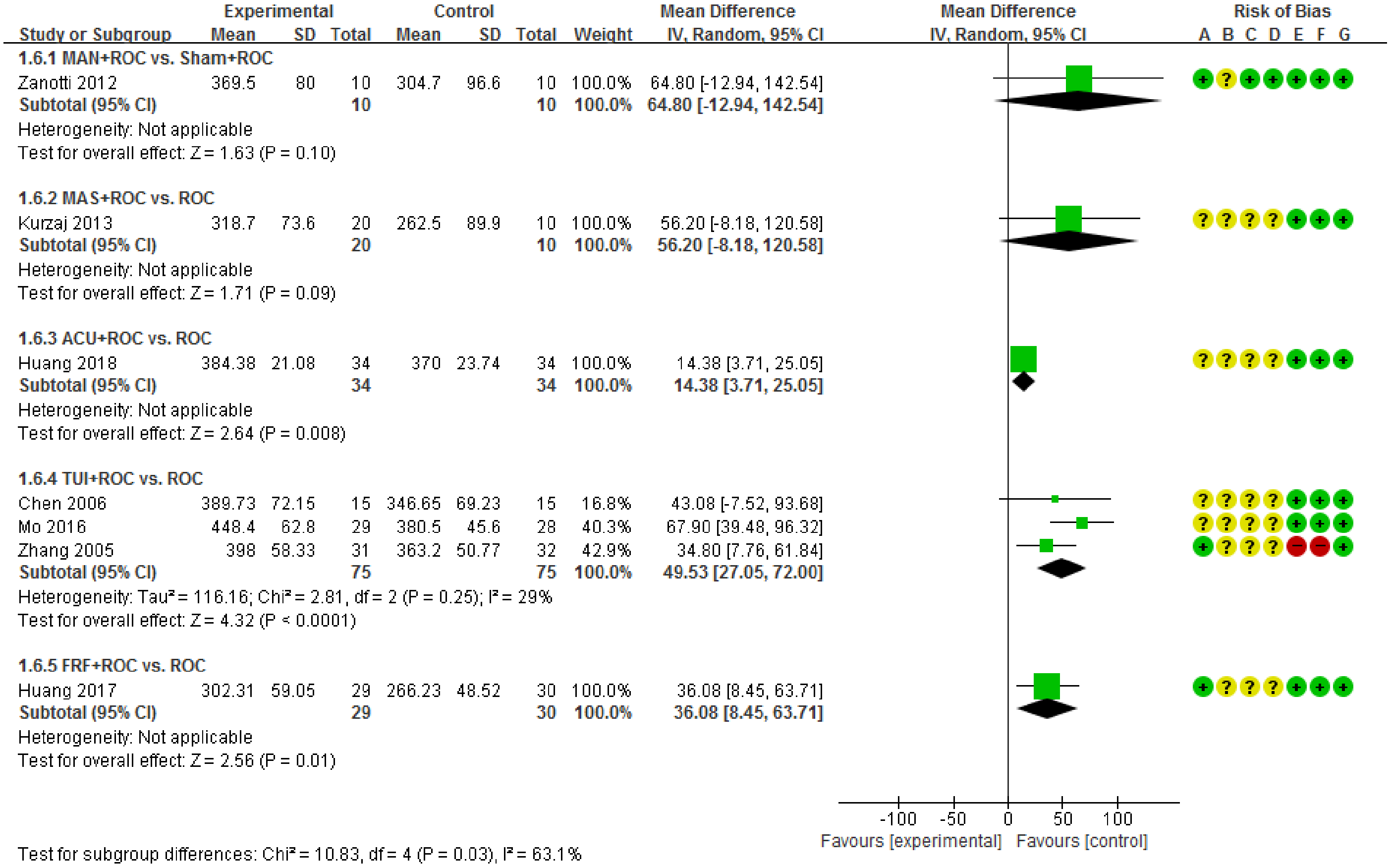

Test for subgroup differences: Chi² = 10.83, df = 4 (P = 0.03), I² = 63.1%

Risk of bias legend
(A) Random sequence generation (selection bias)
(B) Allocation concealment (selection bias)
(C) Blinding of participants and personnel (performance bias)
(D) Blinding of outcome assessment (detection bias)
(E) Incomplete outcome data (attrition bias)
(F) Selective reporting (reporting bias)
(G) Other bias

5. TER (respiratory symptom)

Study or Subgroup	Experimental Events	Experimental Total	Control Events	Control Total	Weight	Risk Ratio M-H, Random, 95% CI	A	B	C	D	E	F	G
**1.7.1 ACU+ROC vs. ROC**													
Gao 2017a	28	30	22	30	10.0%	1.27 [1.01, 1.61]	+	?	?	?	+	+	+
Gao 2017c	28	30	24	30	13.5%	1.17 [0.95, 1.43]	+	?	?	?	+	−	+
Liu 2004	60	64	54	63	39.3%	1.09 [0.97, 1.23]	?	?	?	?	+	+	+
Xiong 2020	58	60	50	60	37.1%	1.16 [1.03, 1.31]	?	?	?	?	+	−	+
**Subtotal (95% CI)**		**184**		**183**	**100.0%**	**1.14 [1.06, 1.23]**							
Total events	174		150										
Heterogeneity: Tau² = 0.00; Chi² = 1.46, df = 3 (P = 0.69); I² = 0%													
Test for overall effect: Z = 3.55 (P = 0.0004)													
**1.7.2 TUI+ROC vs. ROC**													
Chen 2006	13	15	12	15	22.6%	1.08 [0.79, 1.49]	?	?	?	?	+	+	+
Zhang 2005	26	33	26	33	37.2%	1.00 [0.78, 1.28]	+	?	?	?	−	−	+
Zhang 2015	34	40	28	40	40.2%	1.21 [0.95, 1.55]	?	?	?	?	+	+	+
**Subtotal (95% CI)**		**88**		**88**	**100.0%**	**1.10 [0.94, 1.28]**							
Total events	73		66										
Heterogeneity: Tau² = 0.00; Chi² = 1.21, df = 2 (P = 0.54); I² = 0%													
Test for overall effect: Z = 1.23 (P = 0.22)													
**1.7.3 FRF+ROC vs. ROC**													
Gong 2012	17	30	8	30	100.0%	2.13 [1.09, 4.16]	+	?	?	?	+	+	+
**Subtotal (95% CI)**		**30**		**30**	**100.0%**	**2.13 [1.09, 4.16]**							
Total events	17		8										
Heterogeneity: Not applicable													
Test for overall effect: Z = 2.20 (P = 0.03)													
**1.7.4 MAS+ROC vs. ROC**													
Zhuang 2017	34	35	29	35	100.0%	1.17 [1.00, 1.38]	?	?	?	?	+	+	+
**Subtotal (95% CI)**		**35**		**35**	**100.0%**	**1.17 [1.00, 1.38]**							
Total events	34		29										
Heterogeneity: Not applicable													
Test for overall effect: Z = 1.94 (P = 0.05)													

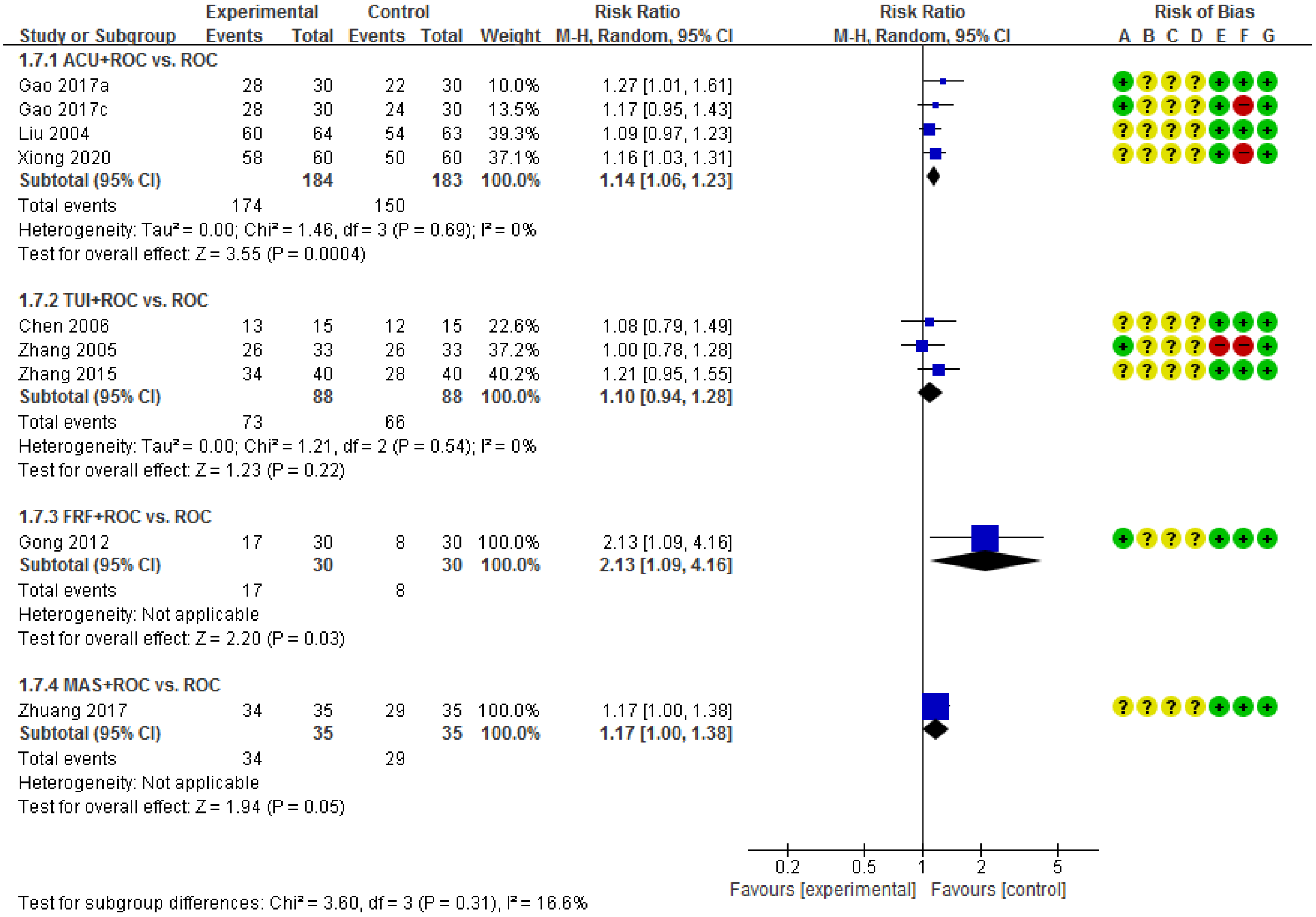

Test for subgroup differences: Chi² = 3.60, df = 3 (P = 0.31), I² = 16.6%

Risk of bias legend

(A) Random sequence generation (selection bias)
(B) Allocation concealment (selection bias)
(C) Blinding of participants and personnel (performance bias)
(D) Blinding of outcome assessment (detection bias)
(E) Incomplete outcome data (attrition bias)
(F) Selective reporting (reporting bias)
(G) Other bias

6. Sputum secretion (mL)

Study or Subgroup	Experimental Mean	Experimental SD	Experimental Total	Control Mean	Control SD	Control Total	Weight	Mean Difference IV, Random, 95% CI	Risk of Bias A	B	C	D	E	F	G
**1.10.1 ACU+ROC vs. ROC**															
Gao 2017b	25.5	2.8	30	30.3	2.1	30	30.4%	-4.80 [-6.05, -3.55]	+	?	?	?	+	−	+
Xiong 2020	24.9	2.4	60	30.6	2.7	60	57.1%	-5.70 [-6.61, -4.79]	?	?	?	?	+	−	+
Zhao 2017	24.5	3.8	29	29.3	3.8	29	12.5%	-4.80 [-6.76, -2.84]	?	?	?	?	+	−	+
**Subtotal (95% CI)**			**119**			**119**	**100.0%**	**-5.31 [-6.00, -4.62]**							
Heterogeneity: Tau² = 0.00; Chi² = 1.60, df = 2 (P = 0.45); I² = 0%															
Test for overall effect: Z = 15.08 (P < 0.00001)															
**1.10.2 MANP+ROC vs .MECP+ROC**															
Wang 2009	20	7.9	60	45.1	8.5	60	100.0%	-25.10 [-28.04, -22.16]	?	?	?	?	+	+	+
**Subtotal (95% CI)**			**60**			**60**	**100.0%**	**-25.10 [-28.04, -22.16]**							
Heterogeneity: Not applicable															
Test for overall effect: Z = 16.75 (P < 0.00001)															

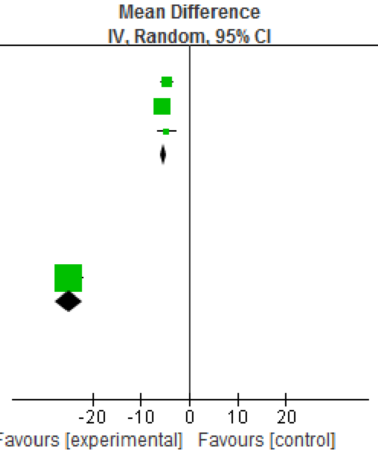

Test for subgroup differences: Chi² = 165.29, df = 1 (P < 0.00001), I² = 99.4%

Risk of bias legend
(A) Random sequence generation (selection bias)
(B) Allocation concealment (selection bias)
(C) Blinding of participants and personnel (performance bias)
(D) Blinding of outcome assessment (detection bias)
(E) Incomplete outcome data (attrition bias)
(F) Selective reporting (reporting bias)
(G) Other bias

7. SpO2 (%)

	Experimental			Control				Mean Difference	Risk of Bias						
Study or Subgroup	Mean	SD	Total	Mean	SD	Total	Weight	IV, Random, 95% CI	A	B	C	D	E	F	G
**1.11.1 ACU+ROC vs. ROC**															
Gao 2017b	97.3	4.1	30	93.3	6.4	30	32.6%	4.00 [1.28, 6.72]	+	?	?	?	+	−	+
Zhao 2017	98.3	5.11	29	95.3	4.11	29	33.1%	3.00 [0.61, 5.39]	?	?	?	?	+	−	+
**Subtotal (95% CI)**			**59**			**59**	**65.7%**	**3.44 [1.64, 5.23]**							
Heterogeneity: Tau² = 0.00; Chi² = 0.29, df = 1 (P = 0.59); I² = 0%															
Test for overall effect: Z = 3.75 (P = 0.0002)															
**1.11.2 MANP+ROC vs .MECP+ROC**															
Wang 2009	91.2	3.8	60	96.2	2.5	60	34.3%	-5.00 [-6.15, -3.85]	?	?	?	?	+	+	+
**Subtotal (95% CI)**			**60**			**60**	**34.3%**	**-5.00 [-6.15, -3.85]**							
Heterogeneity: Not applicable															
Test for overall effect: Z = 8.51 (P < 0.00001)															
**Total (95% CI)**			**119**			**119**	**100.0%**	**0.58 [-5.88, 7.05]**							
Heterogeneity: Tau² = 31.39; Chi² = 60.46, df = 2 (P < 0.00001); I² = 97%															
Test for overall effect: Z = 0.18 (P = 0.86)															
Test for subgroup differences: Chi² = 60.16, df = 1 (P < 0.00001), I² = 98.3%															

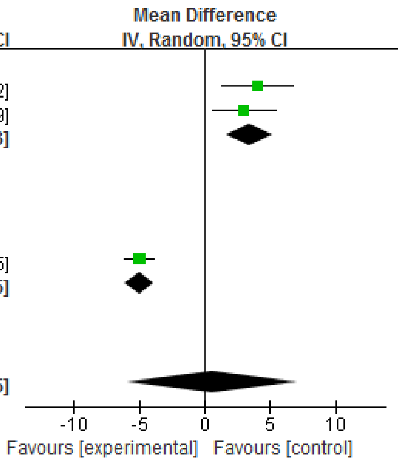

Risk of bias legend
(A) Random sequence generation (selection bias)
(B) Allocation concealment (selection bias)
(C) Blinding of participants and personnel (performance bias)
(D) Blinding of outcome assessment (detection bias)
(E) Incomplete outcome data (attrition bias)
(F) Selective reporting (reporting bias)
(G) Other bias

8. PaO2 (mmHg)

Study or Subgroup	Experimental Mean	Experimental SD	Experimental Total	Control Mean	Control SD	Control Total	Weight	Mean Difference IV, Random, 95% CI	Risk of Bias A	B	C	D	E	F	G
**1.12.1 ACU+ROC vs. ROC**															
Gao 2017a	86.78	9.08	30	67.36	7.58	30	18.9%	19.42 [15.19, 23.65]	+	?	?	?	+	+	+
Gao 2017b	80.8	5.14	30	65.34	5.53	30	21.3%	15.46 [12.76, 18.16]	+	?	?	?	+	−	+
Gao 2017c	80.8	2.16	30	71.38	1.24	30	23.1%	9.42 [8.53, 10.31]	+	?	?	?	+	−	+
Liu 2004	68.78	18.6	64	62.56	16.95	63	15.5%	6.22 [0.03, 12.41]	?	?	?	?	+	+	+
Zhao 2017	80.7	5.24	29	65.24	5.63	29	21.2%	15.46 [12.66, 18.26]	?	?	?	?	+	−	+
**Subtotal (95% CI)**			**183**			**182**	**100.0%**	**13.38 [9.16, 17.60]**							

Heterogeneity: Tau² = 19.84; Chi² = 49.27, df = 4 (P < 0.00001); I² = 92%
Test for overall effect: Z = 6.22 (P < 0.00001)

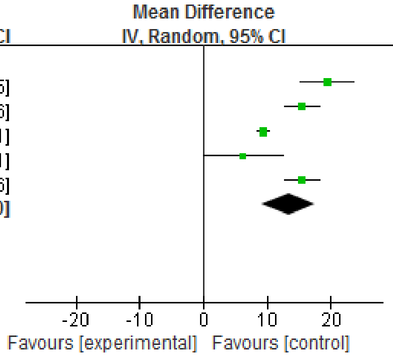

Test for subgroup differences: Not applicable

Risk of bias legend
(A) Random sequence generation (selection bias)
(B) Allocation concealment (selection bias)
(C) Blinding of participants and personnel (performance bias)
(D) Blinding of outcome assessment (detection bias)
(E) Incomplete outcome data (attrition bias)
(F) Selective reporting (reporting bias)
(G) Other bias

9. PaCO2 (mmHg)

Study or Subgroup	Experimental Mean	Experimental SD	Experimental Total	Control Mean	Control SD	Control Total	Weight	Mean Difference IV, Random, 95% CI	A	B	C	D	E	F	G
**1.13.1 ACU+ROC vs. ROC**															
Gao 2017a	50.93	7.43	30	66.38	6.9	30	20.2%	-15.45 [-19.08, -11.82]	+	?	?	?	+	+	+
Gao 2017b	57.57	5.42	30	65.35	6.91	30	21.6%	-7.78 [-10.92, -4.64]	+	?	?	?	+	−	+
Gao 2017c	50.38	6.67	30	58.42	4.89	30	22.1%	-8.04 [-11.00, -5.08]	+	?	?	?	+	−	+
Liu 2004	43.88	16.2	64	48.45	15.83	63	14.9%	-4.57 [-10.14, 1.00]	?	?	?	?	+	+	+
Zhao 2017	57.47	5.52	29	65.25	7.01	29	21.3%	-7.78 [-11.03, -4.53]	?	?	?	?	+	−	+
**Subtotal (95% CI)**			**183**			**182**	**100.0%**	**-8.91 [-12.09, -5.72]**							

Heterogeneity: Tau² = 9.69; Chi² = 16.09, df = 4 (P = 0.003); I² = 75%
Test for overall effect: Z = 5.48 (P < 0.00001)

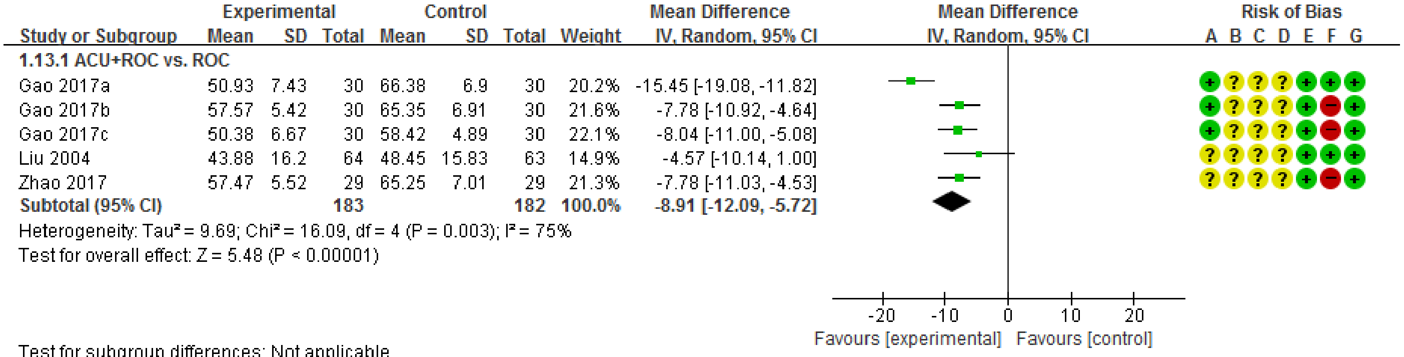

Test for subgroup differences: Not applicable

Risk of bias legend
(A) Random sequence generation (selection bias)
(B) Allocation concealment (selection bias)
(C) Blinding of participants and personnel (performance bias)
(D) Blinding of outcome assessment (detection bias)
(E) Incomplete outcome data (attrition bias)
(F) Selective reporting (reporting bias)
(G) Other bias

10. SaO2 (%)

	Experimental			Control				Mean Difference	Risk of Bias						
Study or Subgroup	Mean	SD	Total	Mean	SD	Total	Weight	IV, Random, 95% CI	A	B	C	D	E	F	G
**1.14.1 ACU+ROC vs. ROC**															
Gao 2017a	96	3	30	89	6	30	46.1%	7.00 [4.60, 9.40]	+	?	?	?	+	+	+
Gao 2017c	98.2	1.41	30	87.3	2.72	30	53.9%	10.90 [9.80, 12.00]	+	?	?	?	+	−	+
**Subtotal (95% CI)**			**60**			**60**	**100.0%**	**9.10 [5.29, 12.91]**							

Heterogeneity: Tau² = 6.70; Chi² = 8.39, df = 1 (P = 0.004); I² = 88%
Test for overall effect: Z = 4.68 (P < 0.00001)

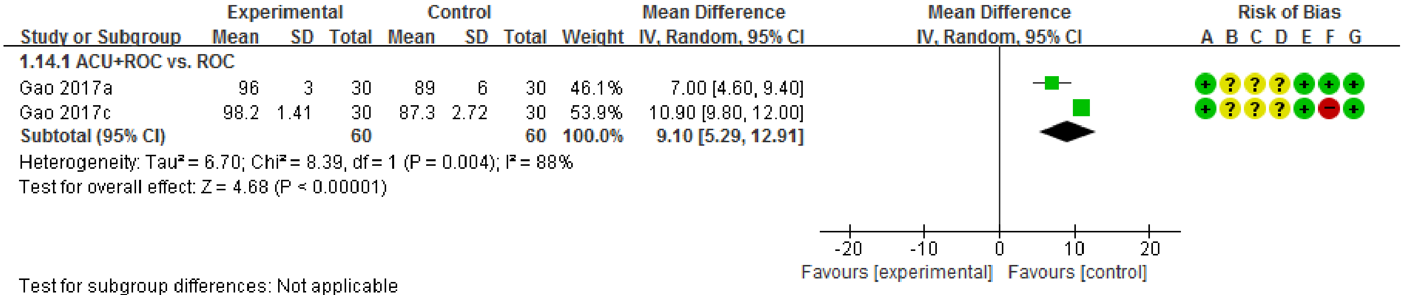

Test for subgroup differences: Not applicable

Risk of bias legend
(A) Random sequence generation (selection bias)
(B) Allocation concealment (selection bias)
(C) Blinding of participants and personnel (performance bias)
(D) Blinding of outcome assessment (detection bias)
(E) Incomplete outcome data (attrition bias)
(F) Selective reporting (reporting bias)
(G) Other bias

11. Adverse events (incidence)

Study or Subgroup	Experimental Events	Experimental Total	Control Events	Control Total	Weight	Risk Ratio M-H, Random, 95% CI	Risk of Bias A	B	C	D	E	F	G
**1.16.1 MAN+ROC vs. Sham+ROC**													
Maskey-Warzechowska 2019	0	19	0	19		Not estimable	+	?	−	?	+	+	−
Noll 2008	2	17	4	17	100.0%	0.50 [0.11, 2.38]	?	?	−	+	+	+	+
Zanotti 2012	0	10	0	10		Not estimable	+	?	+	+	+	+	+
**Subtotal (95% CI)**		**46**		**46**	**100.0%**	**0.50 [0.11, 2.38]**							
Total events	2		4										
Heterogeneity: Not applicable													
Test for overall effect: Z = 0.87 (P = 0.38)													
**1.16.2 Manual chest technique+ROC vs. ROC**													
Cross 2012	15	186	0	186	100.0%	31.00 [1.87, 514.32]	?	?	?	+	+	+	+
**Subtotal (95% CI)**		**186**		**186**	**100.0%**	**31.00 [1.87, 514.32]**							
Total events	15		0										
Heterogeneity: Not applicable													
Test for overall effect: Z = 2.40 (P = 0.02)													
**1.16.3 MAS+ROC vs. ROC**													
Engel 2016	2	8	0	15	100.0%	8.89 [0.48, 165.55]	?	+	?	+	+	+	+
**Subtotal (95% CI)**		**8**		**15**	**100.0%**	**8.89 [0.48, 165.55]**							
Total events	2		0										
Heterogeneity: Not applicable													
Test for overall effect: Z = 1.46 (P = 0.14)													
**1.16.4 TUI+ROC vs. ROC**													
Mo 2016	0	29	0	28		Not estimable	?	?	?	?	+	+	+
**Subtotal (95% CI)**		**29**		**28**		**Not estimable**							
Total events	0		0										
Heterogeneity: Not applicable													
Test for overall effect: Not applicable													

Risk Ratio
M-H, Random, 95% CI

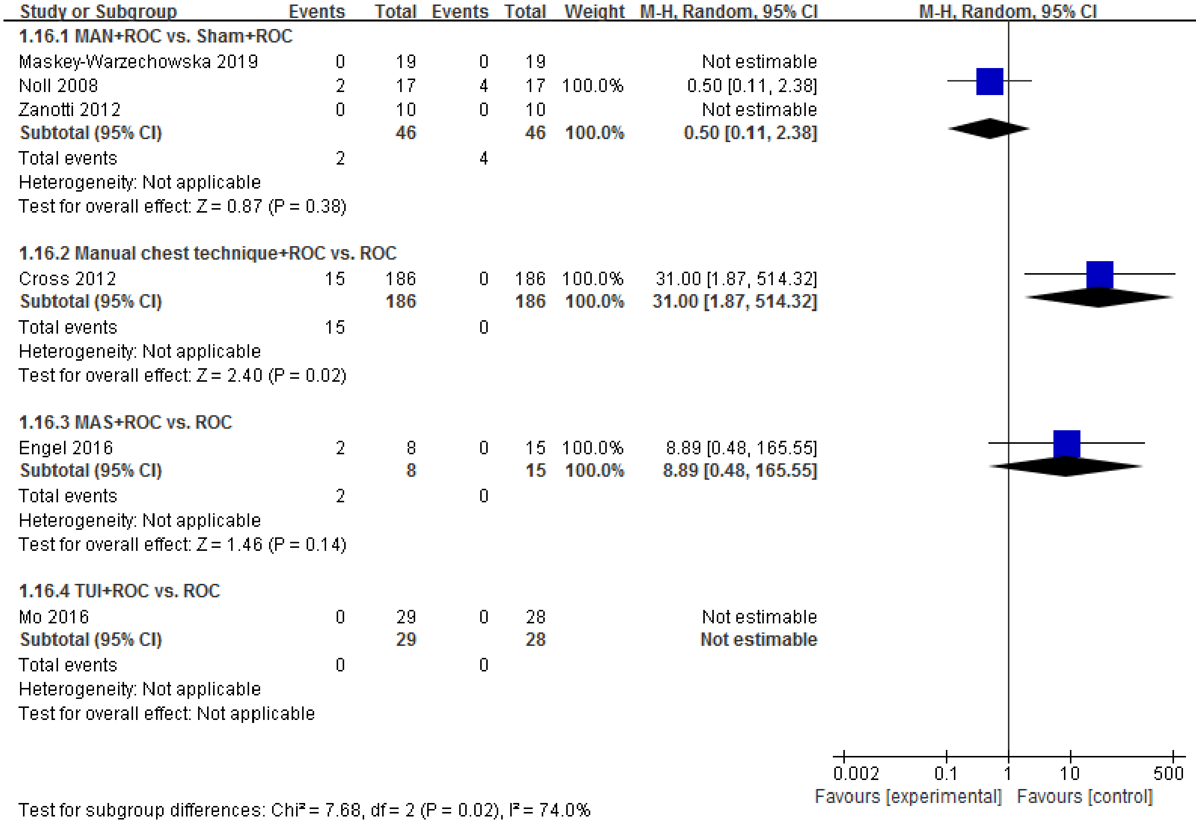

Test for subgroup differences: Chi² = 7.68, df = 2 (P = 0.02), I² = 74.0%

Risk of bias legend
(A) Random sequence generation (selection bias)
(B) Allocation concealment (selection bias)
(C) Blinding of participants and personnel (performance bias)
(D) Blinding of outcome assessment (detection bias)
(E) Incomplete outcome data (attrition bias)
(F) Selective reporting (reporting bias)
(G) Other bias
